# Pathway to Hope: an indigenous approach to healing child sexual abuse

**DOI:** 10.3402/ijch.v72i0.21067

**Published:** 2013-08-05

**Authors:** Diane Payne, Kimber Olson, Jared W. Parrish

**Affiliations:** 1 Justice for Native Children, Chugiak, Alaska, USA; 2 Changing Tides Child and Family Enrichment Center, Anchorage, Alaska, USA; 3 University of North Carolina at Chapel Hill - Injury Prevention Research Center, Chapel Hill, North Carolina, USA

**Keywords:** indigenous, historical trauma, maltreatment, sexual abuse, multigenerational, genocide, traditional, healing

## Abstract

**Background:**

The Alaska Native (AN) population has endured multiple historical traumatic events. This population has poorer health outcomes on nearly all factors compared with Alaska non-Natives with more than 75% reportedly being physically assaulted in their lifetime, and child sexual abuse nearly 6 times the national average.

**Objective:**

This article describes the Pathway to Hope (PTH) program, which is an indigenous approach to ending silence and denial related to child sexual abuse and encourages multigenerational healing.

**Design:**

PTH was developed by ANs who believe that each community is unique, thus strategies for ending denial and support for healing must be woven from the historical context, cultural strengths of individual communities. Strengths-based solutions built on truth, honesty, compassion and shared responsibility for healing and protecting today's children have been profound and successful.

The PTH curriculum addresses child sexual abuse from a historical perspective; that the higher rates of sexual abuse among certain Tribes, regions and communities is linked in part to years of victimisation, but may also be perpetuated by internalised oppression and lateral violence among Tribal members.

**Results:**

Data suggest that community-based dialogue and wisdom of Native elders and spiritual leaders paired with readiness of community service providers are necessary for sustained change. At all levels, this Indigenous model for learning, sharing, helping and healing brings hope for an end to denial and silence about child sexual abuse for Native people.

**Conclusion:**

The PTH program utilises the wisdom and values that have sustained Native people for generations. Ending silence and denial about child sexual abuse and building upon strengths have assisted many Indigenous communities begin the journey toward wellness. Through the PTH, communities have taken steps to accept the challenges associated with establishing safety for children, supporting child victims in healing and to holding offenders accountable.

Pathway to Hope (PTH) is an indigenous approach to ending silence and denial related to child sexual abuse and encourages multigenerational healing in the midst of a myriad of challenges in rural Alaska.

The Alaska Native (AN) and American Indian populations make up approximately 16% of Alaska's population (1). The AN populations have a younger average age relative to the US population (26.9% vs. 35.4%), have less married households (39.1% vs. 52.5%) and have a greater proportion living below the poverty line (19.2% vs. 12.4%) (1). Research further indicates that 1 out of every 3 AN women will be raped and more than 75% will be physically assaulted in their lifetime (2), and child sexual abuse is nearly 3 times the national average (3). In 2007, Alaska's suicide rate was 21.8 per 100,000 people and 35.1 per 100,000 among AN populations and only 11.5 per 100,000 people nationwide (4).

Child sexual abuse is generally correlated to isolation, increased violence, interference with identity and loss of core values among indigenous people. PTH was developed by and for AN communities with an understanding that each community is unique, thus strategies for ending denial and support for healing must be woven with fibres from acknowledging historical context, cultural strengths, individual and community vision as well as support from varied allies. Strengths-based solutions built on truth, honesty, compassion and shared responsibility for healing and protecting today's children have been both profound and successful.

## Intergenerational trauma

The theory of intergeneration trauma seeks to understand why the effects of certain adverse life events transmit to subsequent generations (5, 6). Intergenerational transmission of trauma was first described among Holocaust survivors and their children (5, 7) and has since been studied and identified in other populations (8, 9), with most recent interest among the AN populations (10). Defining and describing historical trauma in the AN populations include the cumulative and collective effects of negative events and assimilation into the contextual structure of the society which are transmitted to subsequent generations (6). Further research explores the context in which the trauma occurs, where the imbalance of power and social injustices exacerbate and perpetuate the transmitting of societal adverse events onto subsequent generations (11, 12).

Although limited empirical research is available on the direct effects of historical trauma, as noted by Sotero, the theory has strong basis in and builds upon 3 tested theoretical frameworks in social epidemiology (psychosocial theory, political/economic theory, social/ecological systems theory) (13, 14).

The AN population has endured multiple historical traumatic events such as, genocides, pandemics, sexual abuse and others (15, 16). This population has poorer health outcomes on nearly all factors compared with Alaska non-Natives (17). Various models and interventions have been used to address these disparities with mixed effects (15). For example, the Home Visitation model designed to prevent child abuse has been effective in reducing child abuse in some settings, but in Alaska was found to have no impact on overall rates (18). Models used without accommodation for cultural and geographical adjustments and those dependent on intensive resources are difficult to assimilate (15, 18). Furthermore, empirical evaluations suggest that Western research may be an imposition, or at times, a deterrent to the intervention outcome and can recapitulate the trauma of colonialism, resulting in further oppression and marginalisation (19). Due to the history of oppression, marginalisation and prejudice, which is not entirely eradicated, ANs may also be reticent to work with people of non-Native cultures.

The PTH curriculum provides a forum for dialogue about historical impacts on current conduct, including safety and abuse of children and holding adults accountable for violent and abusive conduct. Understanding and naming the roots of historical intergenerational trauma through the sharing, supporting, teaching, nurturing techniques used in a train-the-trainer format has been successful in helping Native communities begin to heal from intergenerational traumas at many levels.

## Adverse childhood experiences

The adverse childhood experiences (ACE) study has demonstrated that as the number of ACE increase, negative adult functioning and overall adult health decrease. ACE include verbal, physical, or sexual abuse; domestic violence; parental divorce; and the incidence of family members who are incarcerated, mentally ill, or substance-abusing (20). Increased physical and mental health problems, suicide and other forms of violence have been noted among those reporting a history of childhood sexual abuse (21, 22).

The AN people's experiences appear to be constant with other ACE research. The impact of sexual abuse is known to increase the risk of multiple negative health events (23–26), which can all lead to further victimisation. Like the ACE study, research addressing sexual abuse documents that the severity of abuse correlates with the severity of symptoms and is rarely a stand-alone traumatic event (27, 28).

The research of epigenetics (29) suggests that child experiences, starting in the womb through to the age of 3–5 years can change the expression of human genes (30). Converging evidence from the fields of epidemiology and neurobiology now show that adverse experiences occurring in the first 5 years of life can cause enduring brain dysfunction, dysregulation of affect, difficulty calming the nervous system and difficulties with cognitive functioning which in turn affects the quality of physical and mental health and relationship functioning throughout the lifespan (29, 31, 32), thus explaining, at least in part, why ACE cause later adult impairment (33).

## Relationship of PTH curriculum with intergeneration trauma and ACE

The PTH curriculum addresses child sexual abuse from a historical trauma lens, noting, for instance, that the higher rates of sexual abuse among certain Tribes, regions and communities is linked in part to years of oppression, marginalisation and attempted genocide (34), but may also be perpetuated by internalised oppression and lateral violence among Tribal members.

Mikolajczak et al. demonstrated that increasing the use of alternative, adaptive coping skills could lead to a decrease and, ideally, a cessation of self-harm (35). The developers of the PTH program believe that stripping the survival skills (self-harm and other negative coping skills) away from persons without first introducing positive and helpful adaptive coping strategies will ultimately fail. Evidence-based practice (EBP) and evidence-based treatment (EBT) are today's standard for the delivery of trauma service. Kazdin describes EBP as clinical activities that are informed by evidence about interventions, clinical expertise, and patient needs, values and preferences (36). The cultural aspects of the PTH project are enriched by evidence-based and trauma-informed practises, rather than the other way around (37).

In 2002, Guidelines for International Training in Mental Health and Psychosocial Interventions for Trauma Exposed Populations were published for facilitators working outside their own culture (38). Similar guidance is needed for facilitators teaching AN trauma survivors where the facilitators from outside the community are also stepping into foreign lands with a different cultural landscape. The Guidelines advise that “a deep appreciation of the culture, its historical roots, and the way it has shaped indigenous concepts of mental health and healing” and suggest that local conditions and aspirations be respected so that an integrated model of healing may evolve.

Stories of indigenous approaches to healing body and soul in pre-contact times describe holistic concepts that address both physical and emotional/spiritual aspects of the person. Shaman used plant medicines, prayer, touch, teaching and instruction to support healing when an individual was out of balance and having physical, emotional or spiritual health challenges. The introduction of western religious institutions brought a reduction or elimination of the shaman role in many areas as traditional healing practises were judged as evil. Whether the practise of silence about these travesties is from ancient Native values or the result of severe uninterrupted oppression and extreme isolation, it remains very difficult to have conversations about child sexual abuse and other violence.

Many ANs recognise that a key to holistic wellness is a “strong cultural identification, cultural reclamation, spiritual wellbeing and purposeful living”. Thus, community healing must incorporate cultural accountability and provide a forum for completely different viewpoints and experiences through community-controlled therapeutic services in a bottom-up descriptive fashion (39). The PTH training applies this approach to integration, encouraging participation and sharing of cultural healing methods, consistent with a trauma-informed practises model.

The National Center for Trauma-Informed Care states that the principles of trauma-informed care include understanding trauma and its impact, promoting safety, ensuring cultural competence, supporting consumer control, choice and autonomy, sharing power and governance, integrating care and the belief that healing happens in relationships and recovery is possible (40). These principles are imbedded into the training for PTH.

The PTH model promotes evidenced-based and trauma-informed care from a cultural perspective. It challenges old beliefs and cognitive distortions, and it requires participants to actively engage. While the trainers present facts and teach techniques, participants are asked to complete the structure and anatomy that will form a geographically specific model of culturally-based community healing for each of their own communities.

The PTH trainers offer tools and information from which participants can create a number of intervention and healing possibilities for individuals, groups and families in their own communities. Participants are taught about pacing and containment, encouraged to seek outside counselling when needed and helped to see the painful process of looking inward as a long-term goal that can only be successfully tackled with internal resources and external support. Silencing in the form of historical trauma, continued oppression and internal oppression reinforces trauma narratives and trauma memories, making them more difficult to process and to heal from. This compromises a simple disclosure of trauma in which one experiences cathartic release, which, in isolation, has been found to be re-traumatising, even for many who are not victims of historical trauma (41).

The International Society of Traumatic Stress Studies notes that trauma training, if poorly implemented, may do harm; thus, training should develop the capacities of local leaders, experts and managers, and local organisations to provide on-going support and guidance (42). The PTH model provides for on-going supervision, advanced training and monthly consultation groups for all participants. Unlike models that go into a community, provide the “answers” to community members based on religion, EBPs or theoretical models, PTH aims at an integration of information and practises that can offer the hope of sustainability because it is owned by the participants who expand on it and make it into their own unique and culturally specific model(s) of intervention and healing.

## Historical development of the program

The PTH program was developed through the guidance of a small group of AN people who had been providing victim services and supporting healing through wellness activities in different Tribal communities throughout Alaska. This small group recognised that the core of many issues was decades of individual and community historical trauma much of which was child sexual abuse. Compounding the drastic negative impact of child sexual abuse on individuals was the culture of silence in AN people that stymied healing.

Upon recognition of the far-reaching impacts of child sexual abuse and lack of tools to promote healing, a multigenerational advisory group was convened to guide the creation of a resource that would meet the needs of Tribal communities as well as individuals. The advisory group included respected Native elders, Native victim service providers and young Native adults from diverse Tribal cultures. A 2-day facilitated discussion identified the context for interviews which led to core messages now seen in the PTH video and guidebook. The group also defined the visual and auditory essentials for the video and guidebook to be culturally familiar and effective with Tribal communities. Specifically the advisory group determined that the goals of any intervention must be to: (a) raise awareness about child sexual abuse and the impact of sexual abuse over the lifetime of the child victim; (b) motivate participants to recognise and report suspected sexual abuse, including an awareness of offender behaviors; (c) support healing strategies for child sexual abuse victims and their non-offending family members; and (d) assist Native communities in developing strategies to positively influence community support for child victims by preparing participants to conduct community education about child sexual abuse.

No other programme attempting to promote healing from child sexual abuse in Alaska to date, has originated strictly from the core concepts, values and beliefs of Alaska Indigenous people. Principles of “breaking silence” to heal from various forms of childhood trauma is not unique to Alaska, however the mobilisation of this concept into actionable culturally relevant content are.

The PTH video and curriculum was developed under a grant from the Office for Victims of Crime, US Department of Justice. The curricula and training approach centres on messages in a 2-part video, “Pathway to Hope: Healing Child Sexual Abuse” which was developed over a 2-year period with broad age, gender and experience representation from AN villages. The 40-min video, narrated by a well-known Native actor, shares the voices of more than 45 Native people – both men and women – from diverse communities and cultures, many of whom are child sexual abuse survivors. Interviews were conducted on location at 9 geographically diverse sites around Alaska. A Video Guidebook provides step-by-step guidance on preparation and use of the video to end silence and support child victims, including hand-outs and instructions for interactive activities.

The PTH program is focused on facilitating community dialogue toward ending denial about child sexual abuse, initiating discussion of the issue and providing guidance as the community designs culturally-specific healing strategies. Moreover, this approach provides support for the healing and wellness movement for adult survivors of child sexual abuse, many of whom have only recently begun to talk about the harm they suffered as children. One participant stated: “they (the facilitators) are fine examples of what needs to happen for future healing”.

## Description of the program

The PTH video, guidebook and 3-day community facilitator training are based on the belief that community change and healing can only take place through practises of respecting and honouring the knowledge of Native people and following these guiding principles:

- Indigenous People/Tribal Communities must take responsibility for the safety and healing of children.
- Indigenous People/Tribes must have ownership of social problems as well as the development of solutions to those problems.
- Reclaiming and reviving cultural values, beliefs, practises to heal children and those victimised as children must begin with understanding historical trauma and in multigenerational dialogue.
- On-going mentoring and support for “Indigenous couriers of community change” is essential for Tribal communities to achieve long-term change in attitudes and responses toward children who were victims of sexual abuse.

During the 3-day “Training of Tribal Community Facilitators” session, indigenous learning styles and experiential approaches are used in combination with faculty instructions and guidance on issues relating to child sexual abuse dynamics and victimisation, healing and wellness for indigenous communities and community empowerment strategies. In collaborative interaction, the faculty facilitates participant exploration of established truths, concepts and beliefs (Table I).

**Table I:** Truths, concepts and beliefs explored by facilitators of the PTH

The impact of multigenerational and historical trauma on current safety of children Recognising that there are naturally used protections that prevent us from believing children are sexually abused Holding abusers accountable for their behavior Ending child sexual abuse Examining and understanding how children experience sexual abuse (vulnerability, behaviors, emotional impacts, signs of) Evaluating their own community readiness to end denial about child sexual abuse and begin healing Determining what steps to take toward achieving community ownership in ending silence about child sexual abuse and supporting children Action strategies to toward achieving community ownership to end silence Coming together to celebrate and honour children Teaching adults and children about personal safety Setting community standards about children Healing and support for children when victimised Promoting healing and support for child victims by providing culturally relevant services and education non-offending family members, extended family, and community who will help the child health from both acute and chronic pain

## Breadth and reach of the program

The video and a 2-day training session were first piloted in October 2007. Since the first session, this training has expanded to 3 days and has been presented to more than 270 people working in and for AN communities including Tribal leaders and elders, Tribal victim services, village-based teachers and medical personnel, Alaska State Troopers, Office of Children Services Staff, Village Public Safety Officers, Tribal judges, clergy, community members, child advocacy centre staff, Catholic church representatives, elementary teachers and school staff, Head Start staff and behavioral health providers. In addition, 5 sessions have been provided to more than 120 participants from 18 Tribes in the lower 48 states (Montana, Wyoming, South Dakota and Washington State) since January 2008. Participants at these sessions included highly regarded Traditional Healers, a US Attorney, federal law enforcement officers, as well as many of the same disciplines that were involved in Alaska. In 2013, PTH was provided to Canadian First Nations people representing 12 different Bands, bringing the total to over 400 individuals who have completed the PTH Training of Tribal Community Facilitators curricula. A large percentage of PTH participants have been survivors of childhood sexual abuse or traumas. More sessions are also being planned in Alaska in 2013.

## Process evaluation data and promise of the intervention

Each training event includes the opportunity to gather an inventory of historical trauma events, evaluate the level of community knowledge of child sexual abuse, identify community attitudes about child safety and child sexual abuse and define the community's “helping forces” and “stopping forces” as participants develop strategies for ending denial and silence about child sexual abuse. While these strategies have varied across different events because the community readiness levels are varied, there are some common themes in the goals and action steps identified by PTH participants. In every session, participants noted the need to educate their community members about 3 basic issues: (a) to understand how children are vulnerable to sexual abuse, (b) to recognise the impact of sexual abuse on children and (c) to learn how to support children who report child sexual abuse, so that they can heal while they are still children and not carry the devastating effects into adulthood. Participants are encouraged to specifically design events and set schedules for steps toward community change depending on the community “helping forces” and “stopping forces”. For example, in one community where there were several adult men who had been sexually abused by a priest when they were children, participants designed a “men's wellness support group” and planned to invite a respected elder who is in the PTH video to come and talk with them. That village also decided to provide education for their elders first and to elicit the support of elders to broaden their educational efforts to other groups in the village, including Tribal leaders.

When training events involved participants from several Tribes in a region, strategies usually focus on a different level of change. These groups identified the need to establish inter-tribal public awareness campaigns that they would share and addressing tribal policies through actions such as promoting a “Children's Bill of Rights”, based on one of the activities in the curriculum. One of the regional training events resulted in a commitment, and later grant-writing to establish a child advocacy centre; another regional event helped to bring together the multidisciplinary team members needed to get a new child advocacy centre up and running. In both of these circumstances, the PTH “family” in the region has continued to be a core part of the child sexual abuse response system.

Notable in all of the training events has been the safety for child sexual abuse survivors to speak of their childhood trauma and receive support and an opportunity to heal within their cultural surroundings. Also significant is the breaking down of barriers between agencies, programme and political structures toward creating dependable, child-centred responses to child sexual abuse that will end silence and denial, promote healing and strengthen Tribal communities overall. The universal indigenous cultural foundations of respect, honesty, humility and consideration for others are reclaimed and put into practise with the assistance of the PTH curriculum.

## Conclusion

PTH was first piloted in 2007 with the anticipation that additional Native facilitators could be trained and mentored as faculty. To date, no Native facilitators have taken this role, limiting the number of trainings that can be provided as well as the formulation of new ideas, approaches and processes. Lack of sustainable funding now limits the PTH training to entities that have the resources to pay for faculty costs and fees. The past comprehensive mentoring and support following PTH sessions is no longer available, and updates and revisions to the manual are unfunded. The most significant limitation to PTH has been the lack of a formal programme evaluation. Without a formal evaluation, the programme's effectiveness is limited to qualitative assessments. Despite these limitations, PTH has clearly been in demand and successful.

The PTH program was designed by and for AN people with a goal of drawing from wisdom and values that have sustained Native people for generations, including spiritual beliefs and practise, shared responsibility for the wellbeing of others and resiliency in overcoming adversity while adapting to changing times. These strengths and the desire to stop the pain of abuse, while ending silence and denial about child sexual abuse have helped numerous Indigenous communities begin this part of the journey toward wellness. Through this “training of tribal community facilitators” individuals and communities have taken steps to accept the challenges associated with establishing safety for children, assuring there is support to aid child victims in healing and to hold offenders accountable.

Although each community is different, common threads of community-based dialogue and wisdom of Native elders and spiritual leaders paired with readiness of community service providers, helpers and leaders have emerged. Some resulting changes include the establishment of child advocacy centres, Tribal governments legislating “Children's Bill of Rights” and annual “Protecting and Honoring Our Children” conference. At all levels, this Indigenous model for learning, sharing, helping and healing brings hope for an end to denial and silence about child sexual abuse for Native people in Alaska, and elsewhere.
